# Consequences of over-expression of rat Scavenger Receptor, SR-BI, in an adrenal cell model

**DOI:** 10.1186/1743-7075-3-43

**Published:** 2006-12-15

**Authors:** Eve Reaven, Ann Nomoto, Yuan Cortez, Salman Azhar

**Affiliations:** 1 Geriatric Research, Education, and Clinical Center (GRECC), Department of Veterans Affairs Palo Alto Health Care System, Palo Alto, CA 94304, USA; 2 Digestive Disease Center, Division of Gastroenterology and Hepatology, Stanford University School of Medicine, Stanford, CA 94305, USA

## Abstract

**Background:**

The plasma membrane scavenger receptor, **SR-BI**, mediates the 'selective uptake' process by which cholesteryl esters (CE) from exogenously supplied HDL are taken up by target cells. Recent work suggests that dimer and higher order oligomeric forms of the SR-BI protein are important to this process. SR-BI has been shown to be particularly associated with microvilli and microvillar channels found at the cell surface of steroidogenic cells, and a study with the hormone stimulated adrenal gland has shown impressive changes in the size and complexity of the microvillar compartment as the mass of CE uptake (and accompanying steroidogenesis) fluctuates. In the present study, we examine a cell line in which we overexpress the SR-BI protein to determine if morphological, biochemical and functional events associated with SR-BI in a controlled cell system are similar to those observed in the intact mammalian adrenal which is responsive to systemic factors.

**Methods:**

Y1-BS1 mouse adrenocortical cells were transiently transfected using rat SR-BI-pcDNA6-V5-His, rat SR-BI-pcDNA6-cMyc-His or control pcDNA6-V5-His vector construct using a CaPO_4 _precipitation technique. Twenty four hours after transfection, cells were treated with, or without, Bt_2_cAMP, and SR-BI expression, CE uptake, and steroidogenesis was measured. SR-BI dimerization and cell surface architectural changes were assessed using immunoelectron microscopic techniques.

**Results:**

Overexpression of the scavenger receptor protein, SR-BI, in Y1-BS1 cells results in major alterations in cell surface architecture designed to increase uptake of HDL supplied-CEs. Changes include [1] the formation of crater-like erosions of the surface with multiple double membraned channel structures lining the craters, and [2] dimerized formations of SR-BI lining the newly formed craters and associated double membraned channels.

**Conclusion:**

These data show that overexpression of the scavenger receptor protein, SR-BI (accompanied by suitable hormone treatment and lipoproteins) in susceptible mammalian cells – is associated with increased cholesterol uptake and SR-BI dimerization within a much enlarged and architecturally complex microvillar compartment. These changes duplicate the structural, biochemical and functional changes related to the uptake of HDL CEs normally signaled by the action of ACTH on intact adrenal tissue.

## Background

The selective uptake of cholesteryl esters (CE) from lipoprotein particles such as HDL is a process by which the HDL core-CE is taken into cells without parallel uptake and degradation of the HDL particle itself [1,2]. It represents a major route for the delivery of CEs to steroid producing tissues of rodents and humans [3,4]. Scavenger receptor, class B, type I (SR-BI), a member of the CD36 family of proteins [5], has been identified as an HDL receptor that mediates the uptake of HDL-CEs via this process [3-7], and immunochemical analyses indicate that it is expressed most abundantly in steroidogenic cells and liver [8-13]. Our published data provide evidence that the physical state of the SR-BI protein (i.e., monomer, vs dimeric and higher order oligomeric forms of SR-BI), **and **architectural changes in the cell surface induced by the expression of SR-BI, play major roles in the functional efficiency of the selective pathway [14,15].

Tissues from the rat adrenal gland cortex illustrate these findings particularly well [13]. The microvillar surface of rat adrenal zona fasciculata cells show unusual flexibility and responsiveness to hormonal simulation. In control cells, the entire surface is covered by limp and disorganized appearing microvilli; occasionally the microvilli are upright, occasionally they lie sideways, and every so often the outer external plasma membrane of one microvillus associates with an adjacent microvillus or other plasma membrane surface forming a double membraned channel-like structure [13]. It is such channels where circulating lipoproteins (HDL and LDL) have been shown to be trapped *in vivo *[16,17], and where even small VLDL can often be found [18]. These formations are highly sensitive to hormonal control in the rat adrenal. In ACTH or 17α-E2 treatment of rats there is huge increase in the number of adrenal fasciculata cell microvilli and microvillar channels and substantial architectural changes in the entire microvillar compartment of these cells [13]. There is, as well, a large increment in SR-BI content (adjusted for the obvious increase in microvillar volume) associated with this compartment, and there are corresponding increases in selective HDL-CE uptake. In stark contrast, adrenal tissue from animals in which dexamethasone has been used to down regulate steroidogenesis shows a rapid decline in all these features; as compared to cells from control animals, cortical cell microvilli from dexamethasone treated animals are much reduced in number and complexity, few microvillar channels can be found, SR-BI is virtually gone from the compartment, and selective cholesterol uptake of HDL-CE is barely measurable [13].

In subsequent studies we have shown that these various SR-BI-related changes in the adrenal have a direct relationship to SR-BI dimer formation; i.e., the level of SR-BI dimerization (*i.e. dimers + oligomers of a higher order*) appears invariably associated with the level of selective HDL-CE uptake, SR-BI expression and changes in microvillar compartment architecture in the adrenals of treated animals [14]. A similar relationship between SR-BI dimerization and selective cholesterol uptake has been shown also in cells from other steroidogenic tissues such as the ovary and testis, as well as in a variety of cell lines [15].

In the current study, we set out to learn if the SR-BI related changes observed in the adrenal gland could be reproduced in isolated cells grown in vitro; i.e., does overexpression of SR-BI in such cells lead directly to substantial cell surface membrane changes? We chose as a cell model a mouse adrenal tumor cell line (Y1-BS1 cells), which has certain desirable characteristics: i.e., cells with modest amounts of endogenous SR-BI, yet, like intact steroidogenic tissues, these cells have other essential cellular tools permitting hormone-stimulated steroid hormone (i.e., 20α-dihydroprogesterone) production.

## Methods

### Materials

Iodine-^125^I radionucleotide (carrier free, ~629 GBq/mg; ~17 Ci/mg) was purchased from PerkinElmer NEN^® ^Radionucleotides (Boston, MA). [1α, 2α (N)-^3^H] cholesteryl oleolyl ether (1.78 TBq/mmol; 48.0 Ci/mmol) was obtained from GE Health Care/Amersham Arlington Heights, IL. 20α-Dihydroprogesterone, Bt_2_cAMP, leupetin, PMSF, aprotinin and pepstatin A, goat anti-rabbit IgG-horse-radish peroxidase (HRP) and rabbit-anti mouse IgG-HRP were purchased from Sigma Chemical Co. (St. Louis, MO). The LumiGLO Chemiluminescent Substrate System used in Western blotting was obtained from KPL (Gaithersburg, MD). Goat-anti-mouse IgG coupled to 10 nm colloidal gold and goat anti-rabbit IgG coupled to 15 nm colloidal gold were supplied by Ted Pella, Inc., (Reading, CA). All other reagents used were of analytical grade. Apo E-free high-density hHDL_3 _were isolated as described previously [19]. For uptake and internalization studies, hHDL3 preparations were conjugated with residualizing labels, i.e., ^125^I-labeled dilactitol tyramine ([^125^I]DLT) and [^3^H]cholesteryl oleolyl ether ([^3^H]COE) [20]

### Cell culture and transient transfection

Y1-BS1, a sub-clone of Y1 mouse adrenocortical cells with detectable levels of SR-BI [21] were obtained from Dr. David Williams (SUNY at Stony Brook, NY) in 1998. The cells are normally cultured in Ham's F10 medium supplemented with 15% equine serum, 2.5% fetal bovine serum and 1% penicillin/streptomycin. For transient expression experiments, Y1-BS1 cells were transfected with rat SR-BI-pcDNA6-V5-His, rat SR-BI-pcDNA6-c-Myc-His or control pcDNA6-V5-His vector construct using the CaPO_4 _precipitation technique [22]. Transfection efficiency was determined with β-galactosidase plasmid DNA to be about 10–15% in Y1-BS1 cells. For some studies, 24 h after transfection cells were treated with ± Bt_2_cAMP (2.5 mM) for 24 h. All transfected cells were used for studies 48 h after transfection.

#### Steroidogenic response

Cultures of Y1-BS1 cells were transfected with rSR-BI-V5-pcDNA6.1 (or vector control) constructs for 48 h. Twenty four hours after transfection, some cultures were treated with Bt_2_cAMP (2.5 mM) for an additional 24 h. Subsequently, cells were incubated for 3–5 h in the absence (basal) or presence of Bt_2_cAMP (2.5 mM) ± hHDL_3 _(500 μg protein/ml) as indicated. Following incubation, a suitable aliquot of the medium from each sample was collected, and steroids were extracted from the medium using methylene chloride and quantified by fluorescence in 65% sulfuric acid-35% ethanol using corticosterone as a standard [23].

### Immunoblot analysis

Washed transfected and non-transfected cells were lysed in lysis buffer (125 mM Tris-HCl, pH 6.8, 2% SDS, 5% glycerol, 1% 2-mercaptoethanol, 100 mM PMSF, 10 μg/ml leupeptin, 20 μg/ml aprotinin, and 5 μg/ml pepstatin A). After incubation at 37°C for 15 min, each lysate was sonicated briefly to disrupt chromation (DNA) and then used for immunoblotting.

Aliquots of cell lysates were mixed with equal volumes of 2X Laemmli sample buffer [20 mM Tris-HCl, PH 6.8, 2% SDS (w/v), 10% sucrose (w/v), and 1% 2-mercaptoethanol] and subjected to 7% SDS-PAGE [12]. For each sample, a constant amount of protein (10–40 μg) was loaded on the gel. Protein standards (myosin, 200 kDa; β-galactosidase, 116.3 kDa; phosphorylase b, 97.4 kDa; bovine serum albumin (BSA) 66.2 kDa; and ovalbumin, 45 kDa) were also loaded on the gel. After electrophoretic separation, the proteins were transferred to Immobilon^® ^polyvinyldene difluoride (PVDF) membranes using standard techniques. The protein blots were incubated with either polyclonal rabbit anti-SR-BI or monoclonal anti-V5 for 2 h at room temperature probed with peroxidase-labeled anti-rabbit or anti-mouse IgG and visualized using the LumiGLO Chemiluminescent Substrate System. The resulting autographic chemiluminescence was visualized for different time points (1–10 min), and appropriate films were subjected to densitometric scanning.

### Selective uptake of human HDL_3_-derived CEs

Cultures of Y1-BS1 cells were transfected with rSR-BI-V5-pcDNA6.1 (or vector control) constructs for 48 h. Twenty four hours after transfection, some cultures were treated with Bt_2_cAMP (2.5 mM) for an additional 24 h at 37°C. Cells were incubated with [^125^I] DLT- [^3^H] COE hHDL_3 _(100 μg/ml) for 5 h at 37°C. At the end of incubation, the cells were processed to determine ^125^I-radioactivity, or were extracted with organic solvents and the amounts of CEs and apoproteins internalized via the endocytic and selective pathways were computed [12,21]; results are expressed as the net mass of CEs internalized.

### ImmunoElectron microscopy

a) *Single immunostaining technique*: Y1-BS1 cells transiently transfected with a SR-BI-V5 construct were incubated with Bt_2_cAMP (2.5 mM) for the final 24 h. The cells were harvested, fixed as previously described [14,15], then stained en bloc for 10 min with 0.75% tannic acid prior to embedment in LRGold resin (Ted Pella). Ultrathin sections were immunostained using V5 mAb (40 μg protein/ml) in 1% BSA for 2 h at room temperature followed by secondary antibody staining with goat anti-mouse IgG conjugated to 10 nm gold.

b) *Double immunostaining technique*: Cells were fixed and processed as described above. Ultrathin sections were blocked with 3% BSA and then incubated with a mixture of V5-mAb (40 μg protein/ml) and cMyc-pAb (3 μg protein/ml) in 1% BSA for 2 h at room temperature. Note: to reduce non-specific staining, the polyclonal antibody (anti-cMyc) was pre-absorbed with 10% basal Y1-BS1 cell homogenate. The secondary antibody was a mixture of goat anti-mouse IgG conjugated to 10 nm gold (for V5 staining) and goat anti-rabbit IgG conjugated to 15 nm gold (for cMyc staining).

c) *dimer quantitation*: SR-BI dimers were determined morphologically by estimating the distance between two gold particles considering the lengths of two primary and secondary immunoglobulin molecules and corrected for possible molecule folding [13]. The estimated 'dimer' length between 2 gold particles was calculated to be 2 times the width of a single cross sectional slice of plasma membrane (thus two times the historically accepted value of 100A), and judgment was made on high magnification photographs (36 K × 3) where plasma membrane width was clearly defined.

## Results

### Biochemical/physiological features of Y1-BS1 cells

**Figure 1:**
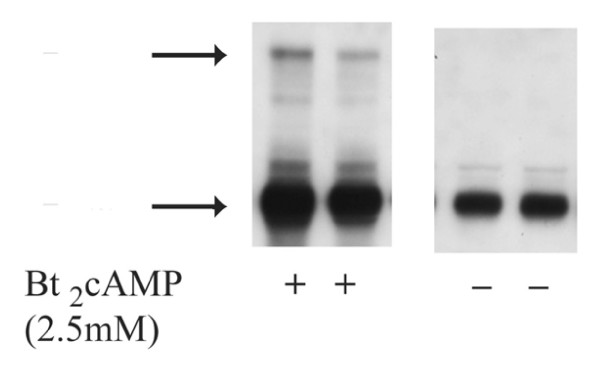
**Western Blot analysis of SR-BI protein**. Cell lysates were prepared from control and Bt_2_cAMP (2.5 mM)-treated Y1-BS1 adrenal cells. Suitable aliquots (10–40 μg protein) were subjected to SDS-PAGE followed by transfer of proteins from gel to Immobiolon^® ^membranes. The blots were incubated with rabbit anti-SR-BI and developed with an HRP-based chemiluminescent detection system. Arrows indicate the position of SR-BI monomer and dimer/oligomers. The approximate molecular weights of top and bottom bands are 160 kDa and 80 kDa, respectively.

Fig. 1 shows the Western blot expression of SR-BI in Y1-BS1 mouse adrenocortical cells under basal conditions and following incubation with Bt_2_cAMP for 24 h. Table 1 compares the **steroidogenic response **of Y1-BS1 cells transiently transfected with empty vector or rat SR-BI and treated with Bt_2_cAMP (2.5 mM) alone, or with hHDL_3_. Whereas the steroidogenic response is similar following hormone (cAMP) treatment of both the vector control and the SR-BI transfected cells, the addition of hHDL_3 _to the vector control cells induces a 2-fold increase in response of the control cells and a 5 fold increase in the transfected cells. It appears, therefore, that additional stores of cholesterol are required for maximal hormone production, and these stores can be supplied by exogenously provided hHDL_3. _Table 2 shows that the **net mass of CE uptake **is, in fact, 2 fold increased in hormone treated Y1-BS1 cells transfected with SR-BI over control (vector) transfected cells.

**Table 1:** Steroid production by control (non-transfected) and SR-BI transfected Y1-BS1 mouse adrenocortical cells

Experimental Conditions	**20α-Dihydroprogesterone **(ng/mg cell protein/5h ± SE)
Control (vector)-transfected Y1-BS1 Cells	
• Bt_2_cAMP (2.5 mM)	4542 ± 990
• Bt_2_cAMP + hHDL_3_	11620 ± 2041
	
*SR-BI *transfected Y1-BS1 cells	
• Bt_2_cAMP (2.5 mM)	5303 ± 1298
• Bt_2_cAMP + hHDL_3_	26340 ± 2059^¶^

Results are Mean ± SE of four separate experiments.

Cultures of Y1-BS1 cells were transfected with rSR-BI-V5-pcDNA6.1 (or vector control) constructs for 48 h. Twenty four hours after transfection, some cultures were treated with Bt_2_cAMP (2.5 mM) for an additional 24 h. Subsequently, cells were incubated for 5 h in the absence (basal) or presence of Bt_2_cAMP (2.5 mM) ± hHDL_3 _(500 μg protein/ml) as indicated. Following incubation, a suitable aliquot of the medium from each sample was collected, and steroids were extracted from the medium using methylene chloride and quantified by fluorescence in 65% sulfuric acid-35% ethanol using corticosterone as a standard.

^¶ ^*p *= 0.0023 *vs *Bt_2_cAMP + hHDL_3_-treated control cells

**Table 2:** Selective HDL-CE uptake by control Y1-BS1 cells and cells transiently overexpressing SR-BI.

Experimental Conditions	**Mass of selective HDL-CE uptake **(ng CE/mg cell protein/5h ± SE)
Control (vector) transfected cells	
• Basal	607 ± 128
• Bt_2_cAMP (2.5 mM)	1802 ± 463^a^
	
*SR-BI *transfected cells	
• Basal	1623 ± 295^b^
• Bt_2_cAMP (2.5 mM)	3514 ± 48^c,d^

Results are Mean ± SE of four separate experiments.

Y1-BS1 mouse adrenocortical cells were transfected with rSR-BI-V5-pcDNA6.1 construct (or control vector) for 48 h. Twenty four hours after transfection, some cultures were treated with Bt_2_cAMP (2.5 mM) for an additional 24 h. Subsequently, cells were incubated with radiolabeled, non-releasable apolipoprotein ([^125^I] DLT) and cholesteryl ester ([^3^H] COE) tags ± Bt_2_AMP (2.5 mM) that would accumulate within cells. Uptake by the selective pathway was determined as described under Experimental Procedures.

^a^*p *= 0.0471 *vs *basal control transfected cells; ^b^*p *= 0.0196 *vs *basal control transfected cells; ^c^*p *= 0.0159 *vs *basal SR-BI transfected cells; ^d^*p *= 0.0434 *vs *Bt_2_cAMP-treated control transfected cells.

Due to the clear cut functional improvement in cholesterol uptake and steroidogenesis following the use of both HDL and cAMP in the SR-BI transfected cells, this treatment was utilized in all subsequent morphological studies utlilizing this cell line.

### Morphological features of control Y1-BS1 cells

Y1-BS1 cells cultured in a medium supplemented with equine serum and fetal calf serum ± stimulation with Bt_2_cAMP are healthy looking cells with a busy cytoplasm and an active cell surface showing occasional patches of microvilli, caveoli, coated vesicles, etc. No unusual cytoplasmic organelles or regions with unusual filamentous activity are seen. Although aliquots of native (non-transfected) Y1-BS1 cells contain substantial amounts of SR-BI as measured by Western blotting (Fig. 1), individual cells immunostained for SR-BI at the electron microscope level show only light SR-BI staining. Treatment with B_2_cAMP in these cells tends to increase surface microvilli and increase SR-BI.

### Morphological changes in Y1-BS1 cells transfected with SR-BI

Y1-BS1 cells are difficult to transfect and despite trials with multiple reagents, transfection with the calcium phosphate precipitation technique was found to be the most efficient. In all cases discussed, B_2_cAMP was used for 24 h prior to cell collection which seemed to produce the most dramatic results.

**Figure 2:**
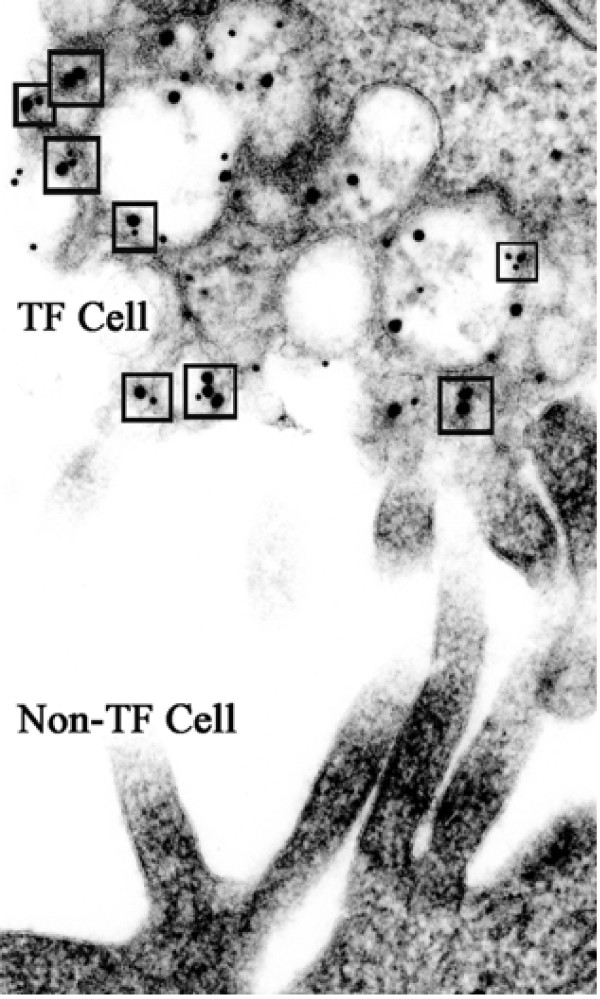
**Photo shows typical surfaces of SR-BI transfected (TF) and non transfected (non-TF) Y1-BS1 cells**. The non-TF cell has a smooth surface, patches of long, slender microvilli, and shows no surface colloidal gold particle representing SR-BI-V5 or -cMyc staining. In contrast, the TF cell has a disrupted surface consisting of vesicles and small vacuoles which are stained with a mixture of gold particles representing V5 staining (small gold) and cMyc staining (large gold). The small boxes identify potential V5/cMyc dimers, where small and large gold particles are in very close contact.

Fig. 2 shows adjacent surfaces of a non-transfected and a double SR-BI construct transfected Y1-BS1 cell immunostained with two SR-BI tags (V5 + cMyc). The non-transfected cell surface shows abundant microvilli, but not one immunogold particle (note: the antibodies used were not for endogenous SR-BI, but specifically for the SR-BI V5 or cMyc tags). In the transfected cell one sees an abnormal appearing vesiculated surface with a high concentration of both large gold (representing antibody to the cMyc tag) plus small gold (representing antibody to the V5 tag) particles. The small gold particles represent staining with a monoclonal antibody to V5 which is extraordinarily specific for the SR-BI-V5 tag. In contrast, the large gold represents a polyclonal antibody to cMyc which, in addition to the tagged portion of the newly transfected SR-BI, may stain other proteins in cells with close homologies to cMyc. In the Y1-BS1 cells used in this study, mitochondria are the prime targets for this non-SR-BI staining with cMyc, and mitochondria from both control and transfected cells in this study show occasional cMyc-gold staining.

**Figure 3:**
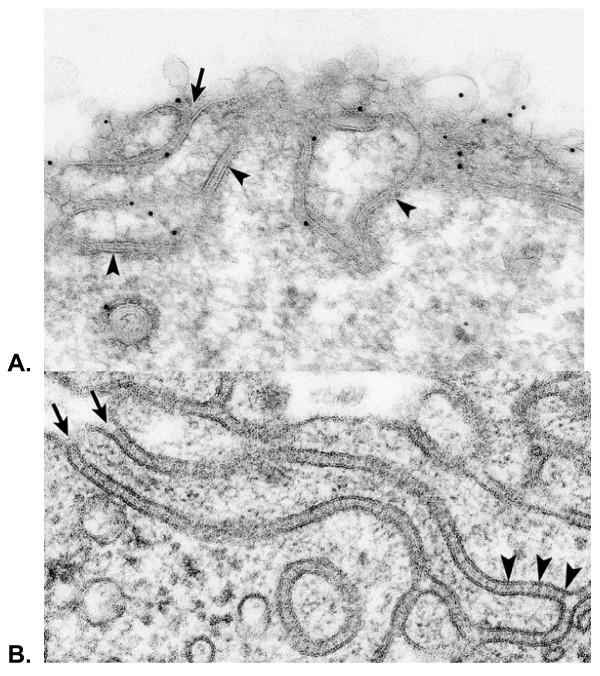
**A – Illustration of another surface membrane pattern of an SR-BI-V5 transfected Y1-BS1 cell revealing undulating double membraned channels (arrowheads)**. Such channel membranes are open to the cell surface (arrow) and dip into the cell where they encircle patches of cytoplasm. **B – The surface of a cortical cell from the adrenal of an ACTH treated rat processed by standard (non-immunochemical) procedures [13]**. The surface of this cell illustrates the similarity between the double membraned surface structures of normal hormone-stimulated adrenocortical cells with those formed in SR-BI transfected cells -as shown in Fig. 3A. Note, that in this normal adrenal cell, the double membraned channels also open to the cell surface (arrows). Striations within the channels (arrowheads) identify lipoproteins [13].

Fig. 3A shows another surface version of an SR-BI transfected Y1-BS1 cell, this time with a convoluted surface of double membraned channels lightly stained for SR-BI-V5. These undulating structures are similar to those observed in adrenocortical cells of ACTH stimulated rats (see Fig. 3B); i.e., cells which express high levels of SR-BI, and where the undulating double membraned channels contain lipoprotein particles [13].

**Figure 4:**
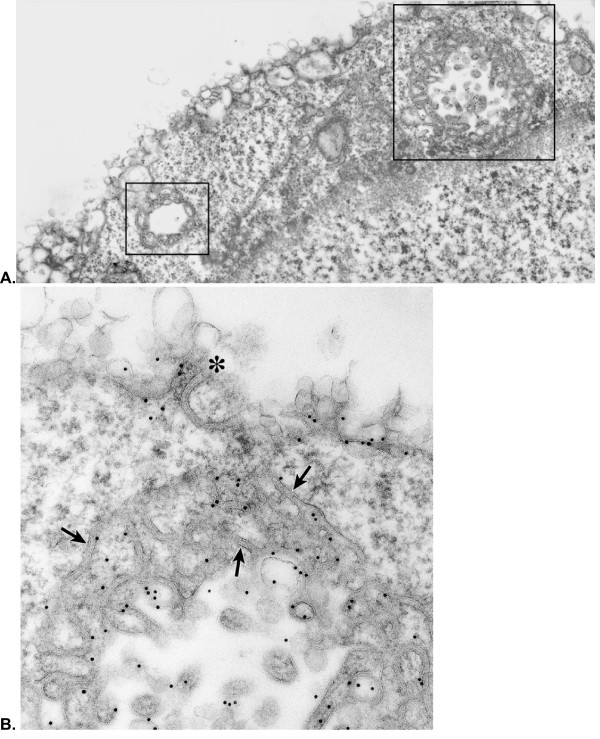
**A – Low magnification photo of a section through a highly expressing SR-BI-V5 transfected Y1-BS1 cell immunostained for the V5 tag**. In addition to the totally disrupted cell surface, note the two complicated crater-like structures (see boxed areas) lined with convoluted double membrane channel structures. **B – A higher magnification photo of the larger crater seen in figure 4A**. Note the center of the mass is gone and just the coiled double membranes (arrows) representing the wall of the crater remain. One loop of coiled membrane opens to the cell surface (asterisk). The gold particles, representing SR-BI-V5 staining, are associated with vesicles and membranes of the crater structure.

Fig. 4A is a low magnification photo of a section through a highly expressing SR-BI-V5 transfected Y1-BS1 cell – immunostained for the V5 antibody. It is clear that the entire surface of the cell is altered. Whereas the majority of the surface looks similar to the changes seen in the transfected cell surface shown in Fig. 2, there are also complex crater-like structures (see boxed areas) with their inner surfaces composed of coiled double membraned channels similar to those seen at the cell surfaces shown in Figs. 3A and 3B. These craters can often be seen to open to the cell surface, the center of the mass is gone, and just the coiled double membrane lining structures remain. Only the surface of the cell and the crater structures are immunogold labeled for SR-BI-V5. One of these craters is shown at higher magnification in Fig. 4B where the connection to the surface is clear, and where it is certain that only the crater membranes (not the surrounding cytoplasm) is immunostained for SR-BI. The double membraned channel forming the continuous wall of this crater has coiled into the surrounding cytoplasm enclosing areas of ordinary cytoplasm including polysomes, coated vesicles and vacuoles.

**Figure 5:**
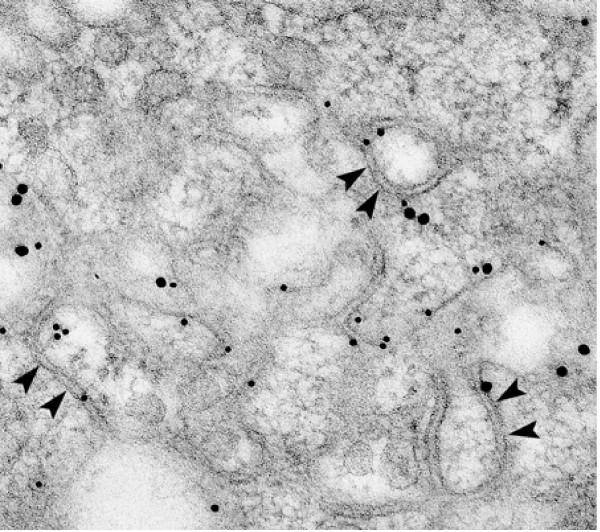
**This figure shows a region with coiled double membranes (arrowheads) found deep within a cell which had been co-transfected with SR-BI-V5 and SR-BI-cMyc cDNA plasmids**. Such sites showing double membraned structures associated with SR-BI constructs are often seen at the edge of vacuoles within the cytoplasm.

In addition to surface disturbances, and double membraned channel structure formation at the cell surface, many SR-BI transfected cells also show areas deep within the cells where immunogold staining reveals patches of double membranes being formed; often such areas appear at the edge of vacuoles (but occasionally there is no obvious connection with a cell structure). In Fig. 5, we show a high magnification photo of such an area found quite deep inside the cell, yet showing the wavy double membraned channels and SR-BI immunostaining similar to that found at the cell surface.

Surprisingly, in the SR-BI transfected Y1-BS1 cells observed in this study, Golgi and endoplasmic reticulum areas were stained only lightly, or not at all.

It is important to note that while all transfected Y1-BS1 cells showed a disrupted surface with a production of SR-BI labeled double membraned channels, not all transfected cells present the same level of change. It is not clear whether the difference between cells reflects the fact that some cells had more time (than other cells) to develop advanced architectural changes, whether we are able to view only a limited slice through any given transfected cell, or whether certain cells are simply more resistant to change than others.

### Biochemical and morphological features of SR-BI dimer formation in SR-BI transfected Y1-BS1 cells

A previous biochemical study from this laboratory identified significant levels of SR-BI monomers in native Y1-BS1 cells, with an increased concentration of monomer SR-BI and some dimer SR-BI expression in cells treated with Bt_2_cAMP [14]. Whereas, the SR-BI monomers are ~3 fold increased in the SR-BI transfected cells, SR-BI dimer/oligomer forms are found in substantially higher proportions relative to their monomer forms in the transfected cells, and are especially high in those cells treated with cAMP (data not shown) – the same category of cells which show increased steroidogenic capacity and selective cholesterol uptake function in table 2 above.

**Figure 6:**
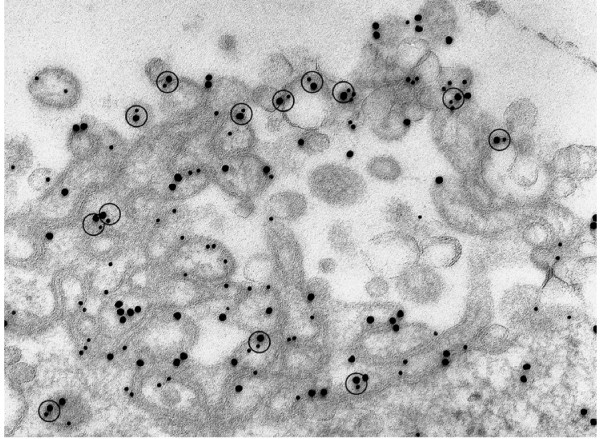
**Heavily immunostained surface crater-like formation with undulating double membranes in a cell co-transfected with SR-BI-V5 and SR-BI-cMyc constructs**. Note: the numerous closely associated gold particles representing dimers of cMyc (large gold), V5 (small gold) and the combination of cMyc + V5 (see circles) with large+small gold.

In the high magnification micrograph of Fig. 6, we see another example of the cell surface double membraned craters described earlier in Fig. 4. In Fig. 6, however, the cell was **co-transfected **with SR-BI V5 (small gold) + SR-BI cMyc (large gold), and immunostained with monoclonal and polyclonal antibodies to the two tags as shown with different sized gold particles. The co-transfection in this case permits identifying SR-BI dimer formation morphologically, and provides the possibility of quantification of dimer and oligomer forms as shown in Table 3. Despite corrective measures, there is always concern about random gold clustering in solutions, and, in our effort to quantitate dimers, the possibility exists that closely associated gold particles may not necessarily represent true dimer formations. As shown in Fig. 6, it is possible to get around this issue by considering 3 possible types of closely associated gold particles (as defined in the Methods section): 2 closely associated *large *gold particles, 2 *small *gold particles, but also, closely associated *large + small *gold particles. In Fig. 6, and in other similar areas of SR-BI stained double membranes used to quantify dimer formation morphologically (Table 3), it is clear that the relative numbers of large + small gold combinations is substantial, and also similar in number to the other possible dimer combinations. This is a reality check: large/small gold dimer combinations indicate that antibodies for two entirely different proteins V5 and cMyc are not only present at the same cell site, but in close enough association to form numerous dimer formations when stained with their respective secondary antibodies tagged with different sized gold particles. Table 3 shows the results of quantifying SR-BI dimer combinations from cell surface related areas of SR-BI immunogold found in co-transfected cells, and indicates that when all the possible dimer formations are considered together, more than 40% of the gold found in these areas represents sites of SR-BI dimerization. SR-BI staining of regions with double membrane formations found *inside *transfected cells (as described in Fig. 5), also show substantial dimerization of SR-BI.

**Table 3:** Immunogold Dimer Formation Associated with SR-BI-enriched Cell Surface Sites of SR-BI-V5 + SR-BI-cMyc Transfected Y1-BS1 Cells

Expt. no.	Total gold in region measured	Large + small heterodimer	Small + small homodimer	Large + large homodimer	Total dimers	Total* dimer gold	Dimer gold as percent of total gold
1	615	42	33	46	121	242	39%
2	1513	99	94	99	292	584	39%
3	756	53	85	32	170	340	45%

*Note: Since each dimer represents two gold particles, "total dimer gold" represents total dimers X 2. This number divided by the "total gold in the region" represents the percentage of total gold found as dimers.

Small 10 nm gold coupled to anti-mouse-IgG was used to identify V5 monoclonal antibody staining of the Y1-BS1 cells transfected with an SR- B1-V5 tag cDNA construct.

Large, 15 nm gold complexed with anti-rabbit IgG was used to identify cMyc polyclonal antibody staining of the Y1-BS1 transfected SR-BI-cMyc tag plasmid DNA. Close contact between gold particles was used to identify the 3 possible dimer combinations, and, as shown here, the numbers were similar (indicating reasonable levels of first and second antibody concentrations had been used to balance differences in antibody sensitivities and size of gold particles).

Large/small gold dimer combinations indicate that secondary antibodies representing anti-mouse IgG gold (V5 staining) and anti-rabbit IgG gold (cMyc staining) are present at the same cell site, and, as such, eliminate the possibility that random gold clustering in solution is responsible for the dimer-like formations.

## Discussion

This study clearly shows that transfection of Y1-BS1 cells (a mouse adrenal cell line) with rat SR-BI using a calcium phosphate precipitation technique results in cells with a dramatically altered cell surface. One version of this disrupted surface can be described as an explosion of SR-BI stained vesicles -where the original plasma membrane (including caveoli, clathrin coated pits, typical microvillar structures) no longer exists, but appears to have become part of the vesicular mass. However, in most of the transfected cells one also sees the development of multiple and quite elaborate SR-BI stained double membraned channel structures at the cell surface, which in their width, their intertwining curving nature, and their general architecture resemble the complex double membraned structures seen associated with the microvillar compartment of hormone-stimulated adrenocortical or ovarian cells in tissues of the normal rat. It is clear that the morphological plasma membrane changes we see are always associated with SR-BI (as they are in intact sections of steroidogenic tissue), and in the case of the Y1-BS1 cell preparations, only cells showing accumulations of SR-BI, show any of the physical changes noted.

In addition to these cell surface related changes, SR-BI transfected cells may also show accumulations of SR-BI intracellularly, often associated with non-identifiable vacuoles or masses found deep within the cell. Along the edges of these masses, patches of SR-BI accumulation can occasionally be identified, and, no matter how big or small the SR-BI mass, it is always associated with double membraned structures – some circular, some long, but in every way identical morphologically to the double membraned channel structures associated with the cell surface. While it is possible that these intracellularly located structures are, in fact, connected to the cell surface (at a point not visible in our sections), it seems possible that these deep sites are where SR-BI has accumulated, but has not been appropriately transported to the cell surface. Perhaps, in such cells, the production of SR-BI is so large that transport and utilization of the protein at the surface does not have sufficient time (or sufficient delivery proteins or equipment) to occur. And, like in SF9 insect cells infected with SR-BII (Reaven & Azhar, unpublished observations), the stalled delivery system deposits the protein at some site, and with the help of available cell machinery, the double membraned channels meant for the surface are actually produced in situ.

How SR-BI is transported through the cell is not yet clear. The multiple glycosylation sites of SR-BI [19] strongly suggest that the nascent protein must pass through *trans *Golgi membranes, but remarkably little SR-BI is visible in, or around, the Golgi areas, despite the use of multiple antibody types (anti SR-BI against the *C*-terminal or extracellular domain (ECD) or V5 or cMYc tagged SR-BI). Indeed, the most heavily transfected cells do not seem to have easily identifiable Golgi areas at all, and one wonders if transport of the newly formed protein has exhausted the Golgi membranes-which then are distributed throughout the cell and perhaps form the basis for some of the intracellular sites of SR-BI which we do see. Likewise, the existing endoplasmic reticulum is not heavily labeled for SR-BI, though in occasional cells one can find patches of wavy ER-like membranes (some even with attached ribosomes) which appear to stain lightly for SR-BI. Perhaps passage of the nascent protein through these traditional membranes is too rapid a process to show up dramatically. Or, as is always possible, the antigenic sites of SR-BI may be masked at these early stages, and it is not until the protein later dimerizes *in situ*, or at the cell surface, that the protein becomes available for immunostaining.

What remains clear is that cellular sites where SR-BI is prominent (e.g. surface membrane + surface or intracellular craters) show a high degree of SR-BI dimerization. Approximately 40% of the gold particles found in such areas are in close contact with each other, and this remains true whether the immuno-gold represents staining of two SR-BI V5 molecules, two SR-BI cMyc molecules, or a combination of the two different tags (therefore a dimer composed of SR-BI-V5+SR-BI cMyc). Indeed, the double tagged dimer re-enforces the idea that SR-BI, whatever its identification tag, migrates to the same cell region, and is intimately involved in the construction of the double membraned channels which result.

Finally, to what extent do the attributes of an exquisitely complex cell surface of double membraned channels composed of highly dimerized molecules of SR-BI lead to heightened cell function? We suggest that cells with coils of double membraned channels containing dimerized SR-BI are capable of taking up vast numbers of HDL particles [13,14] in any given preparation. Such cells with their substantially increased cell surface and heightened ability to attract and trap HDL will also deliver increased CE mass to the cell interior as substrate for increased steroid production.

## Conclusion

These data indicate that overexpression of the scavenger receptor protein, SR-BI, in a receptive mouse adrenocortical tumor cell line (Y1-BS1) leads to a complex cell surface of double membraned channels endowed with highly dimerized molecules of SR-BI. As a result, this hormone-stimulated adrenal tumor cell overexpressing SR-BI, like cells of the ACTH stimulated rat adrenal, is capable of capturing increased numbers of HDL, internalizing increased amounts of cholesteryl esters and secreting increased levels of steroid hormone.

## Abbreviations

CE, Cholesteryl esters; HDL, high-density lipoprotein; LDL, low-density lipoprotein; VLDL, very low-density lipoprotein; SR-BI, scavenger receptor class B, type I; DLT, dilactitol tyramine; COE, cholesteryl oleolyl ether; 17α-E2, 17α-ethinyl estradiol

## Competing interests

The author(s) declare that they have no competing interests.
